# Intraretinal Hyperreflective Foci as an OCT Feature Associated With Retinal Remodeling After Macular Hole Closure

**DOI:** 10.1167/iovs.67.12.9

**Published:** 2026-10-05

**Authors:** Nicola Valsecchi, Giuseppe Gregorio Fusca, Luigi Apa, Francesca Del Monte, Claudio Scardovelli Fava, Alessandro Finzi, Antonio Moramarco, Maurizio Mete, Luigi Fontana

**Affiliations:** 1 Ophthalmology Unit, Dipartimento di Scienze Mediche e Chirurgiche, Alma Mater Studiorum University of Bologna, Bologna, Italy; 2 IRCCS Azienda Ospedaliero-Universitaria di Bologna, Bologna, Italy

**Keywords:** macular hole, hyperreflective foci, optical coherence tomography, Müller cells, photoreceptor recovery, surgical healing

## Abstract

**Purpose:**

To investigate intraretinal hyperreflective foci (HF) within the foveola in the healing process following surgical closure of full-thickness macular holes (FTMHs), using lamellar macular holes (LMHs) and vitreomacular traction (VMT) as comparisons.

**Methods:**

Analysis of spectral-domain optical coherence tomography (OCT) scans from 48 FTMH eyes undergoing surgery was conducted, with 14 LMH and 10 VMT eyes serving as controls. HF were defined as dot-like intraretinal lesions with reflectivity similar to the retinal nerve fiber layer and without back-shadowing, showing a vertical distribution pattern within the foveola. Baseline OCT parameters, including minimal linear diameter, basal diameter, hole height, and macular hole index (MHI), defined as the ratio between hole height and basal diameter, were assessed. HF patterns were evaluated in relation to external limiting membrane (ELM) restoration and ellipsoid zone (EZ) integrity after surgery.

**Results:**

Among the 48 FTMH eyes, 43 achieved successful closure (89.6%). HF were identified in all 43 eyes achieving anatomical closure at the first postoperative assessment, compared with 50% in LMH and 50% in VMT controls (*P* < 0.001). HF preceded complete ELM restoration in nine of 43 eyes (20.9%), whereas in the remaining cases both events were detected simultaneously at the same postoperative visit. At final follow-up, HF persisted in 86.0% of eyes and were associated with baseline larger base diameter (*P* = 0.044) and baseline lower macular hole index (MHI; *P* = 0.007). Complete EZ integrity was achieved in 65.1% of eyes and was significantly associated with HF resolution (*P* = 0.047) and better final visual acuity (*P* = 0.038).

**Conclusions:**

HF displaying a vertical pattern within the foveola may represent an early structural OCT feature associated with retinal remodeling after macular hole surgery. Further studies are needed to determine their biological significance and potential role as an imaging marker of macular hole healing.

Macular hole (MH) surgery currently achieves anatomical closure rates exceeding 90%; however, functional recovery remains variable and sometimes unpredictable, reflecting incomplete understanding of the biological mechanisms underlying MH healing.^1^ The unique foveal anatomy may partially explain this variability. In particular, the central fovea contains a specialized group of Müller cells, known as the Müller cell cone, which forms the structural floor of the foveola and stabilizes central cone photoreceptors. Disruption of the Müller cell cone is central to MH pathogenesis, and anatomical restoration of this structure during surgery is essential for achieving functional visual recovery.^2^

The role of gliosis in MH healing is well established. Histopathologic studies by Gass and colleagues^3^ and subsequent investigations^4^ demonstrated that Müller cell proliferation and gliotic repair are key determinants of postoperative MH closure. In vivo optical coherence tomography (OCT) studies supported this hypothesis by showing that glial tissue contributes to healing through transient scaffolding for tissue reorganization.^5^^,^^6^ However, the functional significance of postoperative glial changes remains controversial, with some studies suggesting that glial proliferation facilitates anatomical closure and others implicating excessive gliosis in incomplete photoreceptor recovery and poorer visual outcomes.^4^

Recent OCT findings have provided additional insights into foveal healing dynamics. Scharf et al.^7^ identified a central vertical hyperreflective line persisting after surgical closure, potentially representing the first in vivo visualization of Müller cell organization. Spaide^8^ proposed that MH healing recapitulates developmental foveation and described transient granular hyperreflective foci (HF) preceding photoreceptor restoration. Although HF have been extensively described in diabetic macular edema, retinal vein occlusion, and age-related macular degeneration, their biological significance in MH healing remains undefined.^9^^–^^11^

We hypothesized that foveolar HF may represent an OCT feature associated with structural remodeling during the early phases of MH healing, potentially reflecting active foveal repair after surgical closure. Therefore, the aim of the present study was to characterize the presence and temporal evolution of postoperative HF after pars plana vitrectomy (PPV) with internal limiting membrane (ILM) peeling for full thickness macular holes (FTMHs), and to investigate their association with external limiting membrane (ELM) and ellipsoid zone (EZ) restoration and postoperative visual outcomes. To further explore whether this OCT feature is related to the extent of baseline foveal structural disruption rather than representing a nonspecific postoperative response, FTMH eyes were compared with LMH and VMT eyes, which are characterized by different mechanisms of foveal alteration and variable involvement of the outer retinal layers.

## Methods

### Study Population

This was a retrospective, observational study based on a review of clinical records of consecutive patients affected by stage 2, 3, and 4 MHs who underwent PPV and ILM peeling at the Unit of Ophthalmology, University of Bologna, between January 2022 and January 2025. All patients included in the study underwent baseline assessments, as well as postoperative follow-up examinations within 1 month, 1 to 3 months, 3 to 6 months, 6 to 12 months, and 12 to 18 months after surgery. Exclusion criteria were significant media opacities (other than cataracts), other macular conditions (such as intermediate or late-stage age-related macular degeneration or diabetic macular edema), poor-quality imaging, any prior ocular surgery (except cataract surgery), an axial length greater than 26 mm, and recent trauma in the last 12 months All procedures adhered to the tenets of the Declaration of Helsinki, and all participants provided written informed consent. This study was approved by the Ethics Committee of Bologna, Italy (Cod CE: 53/2025/Oss/AOUBo). A control group of patients who received PPV and ILM peeling for lamellar macular holes (LMHs) and vitreomacular traction (VMT) was also included in the study, with identical inclusion and exclusion criteria applied.

### Ophthalmological Assessment

At baseline and each follow-up visit, the following evaluations were performed: best-corrected visual acuity (BCVA) assessment (in logMAR), slit-lamp examination, intraocular pressure measurement, dilated fundus imaging with a 90-diopter lens, and spectral-domain optical coherence tomography (SD-OCT; SPECTRALIS HRA+OCT; Heidelberg Engineering, Heidelberg, Germany). Axial length was determined at baseline by optical biometry (IOLMaster 700; Carl Zeiss Meditec, Jena, Germany).

### OCT Image Acquisition

All subjects underwent standardized SD-OCT imaging using a dense 20° × 15° raster scan protocol consisting of 145 B-scans spaced 30 µm apart, with additional vertical and horizontal single scans through the foveal center (automatic real-time values ≥ 14). All images were processed with 1:1-mm aspect ratio correction. At baseline, two masked, independent observers (GGF, FDM) classified all OCT scans and measured morphologic parameters in FTMHs including maximum basal diameter, hole height, and MH index (calculated as height/maximum basal diameter). Measurements from the first observer (GGF) were used for primary statistical analysis, and measurements from the second observer (FDM) were retained for intergrader reliability assessment. Furthermore, LMHs were classified as true or degenerative LMHs and tractional LMHs or epiretinal membrane (ERM) foveoschisis, as previously described.^12^

At all postoperative follow-up visits, the same two masked reviewers (GGF, FDM) independently assessed the integrity of the ELM and EZ, documenting the time interval from surgery to each assessment. Both ELM and EZ were evaluated in the foveola (central 350 µm of the fovea) and, when present, were graded in a binary fashion as either complete or incomplete. In cases of disagreement between the two observers, a third masked observer (MM) made the final determination.

### Assessment of HF

The presence of HF was evaluated in all SD-OCT scans obtained after surgery at different follow-ups for MH, LMHs, and VMT. HF were defined as dot-like lesions (>10 µm and <30 µm) with reflectivity similar to the retinal nerve fiber layer and lacking back-shadowing, as previously described.^9^ For the purpose of the present study, HF were assessed within the central 350 µm of the foveola in eyes with restored foveal architecture, where they were required to display a vertical distribution pattern compatible with the anatomical organization of the central foveola. Because the assessment of the vertical foveolar HF pattern relied on the presence of restored foveolar architecture, this classification was applied only to eyes in which the MH was anatomically closed. A minimum of two HF showing a vertical distribution pattern within the assessed region was required for HF classification in order to distinguish this characteristic organization from isolated hyperreflective dots. The foveal area of interest was delineated using the caliper function of the Heidelberg OCT system. Two masked graders assessed the presence of HF (GGF, FDM); in cases of disagreement between the two observers, a third masked observer (MM) made the final determination. Representative cases are shown in Figure 1.

**Figure 1.**
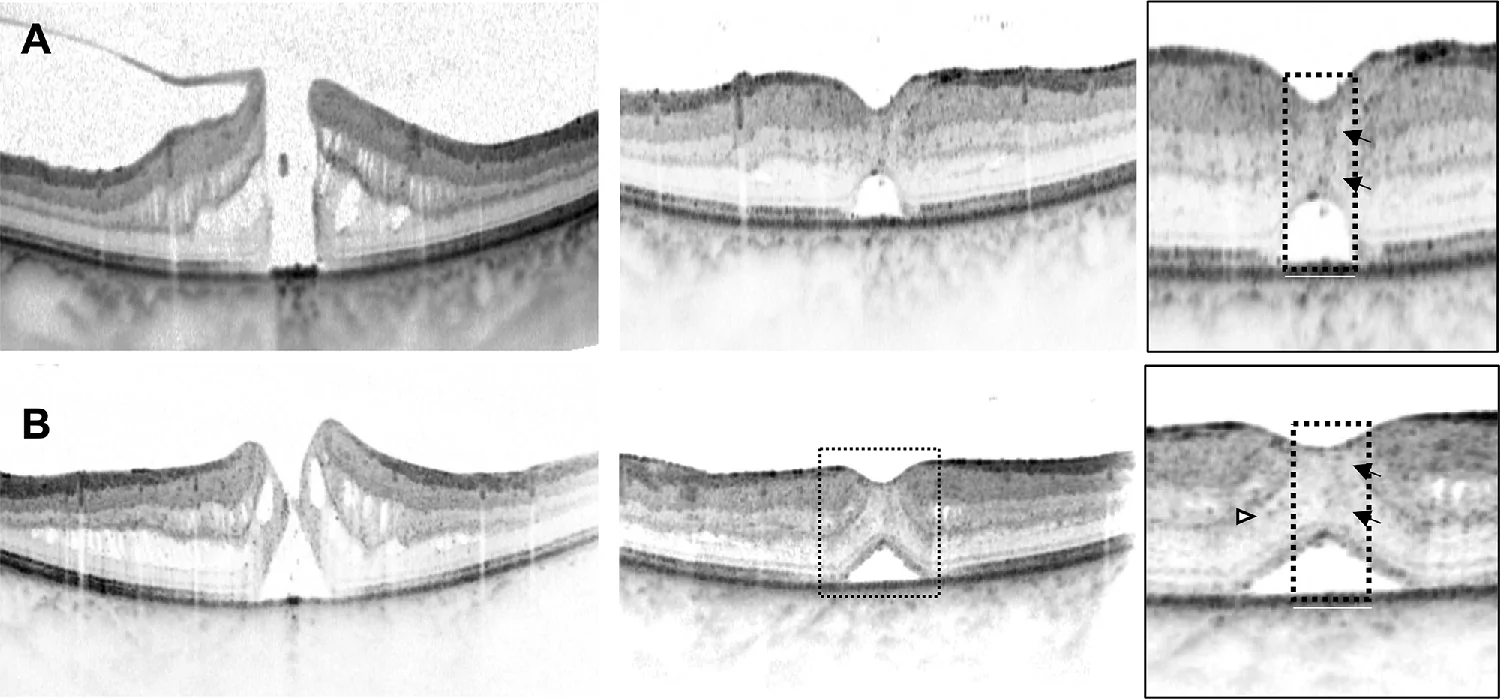
**Assessment of HF with a vertical distribution pattern in the foveola following surgical closure of FTMHs. **(**A**) Preoperative OCT B-scan of a 74-year-old female with FTMH (*left*), OCT B-scan at 3-month follow-up (*center*), and magnified foveolar view (*right*). The central 350-µm region was delineated (*white line*), and HF were evaluated within this region of interest (*black dashed rectangle*). Note the vertical distribution of HF within the foveola (*black arrows*), complete ELM restoration, and absence of the EZ. The two graders were concordant in their assessments. (**B**) Preoperative OCT B-scan of a 64-year-old male with FTMH (*left*), OCT B-scan at 5-month follow-up (*center*), and magnified foveolar view (*right*). The central 350-µm region was delineated (*white line*), and HF were evaluated within this region of interest (*black dashed rectangle*). The two masked graders disagreed on the presence of vertically arranged HF; the third grader adjudicated the assessment as positive for HF (*black arrows*). Note the presence of isolated HF outside the foveolar region (*white arrowhead*).

### Surgical Technique

All patients underwent PPV with ILM peeling. Anesthesia type (general or local) was selected based on patient preference and anesthesiologist recommendation. A 25-gauge vitrectomy was performed using the Constellation platform (Alcon, Fort Worth, TX, USA). In phakic eyes, cataract surgery was performed prior to vitrectomy. Complete vitrectomy with peripheral vitreous base shaving was performed, and posterior hyaloid separation from the retina was obtained as needed. The ILM was peeled from the foveal center to the vascular arcades using microforceps (Grieshaber ILM Forceps; Alcon). Membrane Blue Dual dye (Dutch Ophthalmic Research Center International BV, Zuidland, Netherlands) was applied to the retinal surface to enhance ILM visualization during peeling. Fluid–air–gas exchange was performed at the conclusion of surgery using sulfur hexafluoride (SF_6_) in all cases. All patients received standard postoperative management with topical antibiotics and anti-inflammatory medications. Patients were instructed to maintain a face-down position for at least 5 days following surgery. The same surgical technique was applied for both LMHs and VMT. When an ERM was identified, it was removed during the same procedure.

### Statistical Analysis

Demographic and clinical data were summarized using descriptive statistics, including mean ± standard deviation (SD) or median with interquartile range (IQR) for continuous variables according to data distribution and absolute frequency with percentage for categorical variables. Normality of continuous data was assessed using the Shapiro–Wilk test. Interrater reliability for quantitative assessment of MH parameters at baseline was assessed using the absolute agreement model of the intraclass correlation coefficient (ICC). Also, interrater agreement for HF presence was assessed using Cohen's weighted kappa (κ) coefficient. One-way analysis of variance (ANOVA) was used to compare continuous variables (including age, follow-up duration, and BCVA) across the three study groups (FTMH, LMH, and VMT). Fisher's exact test was used to evaluate associations between categorical variables. Independent-samples *t*-tests were used to compare demographic data, OCT morphometric parameters, and BCVA where appropriate. Comparisons between eyes with persistent versus resolved HF at final follow-up were performed using the Mann–Whitney *U* test because of the limited sample size of the HF resolution subgroup. *P* < 0.05 was considered statistically significant. All statistical analyses were conducted using SPSS Statistics 26 (IBM, Chicago, IL, USA).

## Results

### Study Population and Demographics

Over 155 eyes were assessed, and a total of 72 eyes of 72 patients were included in this study: 48 eyes with FTMHs, 14 eyes with LMHs, and 10 eyes with VMT. Demographic and baseline clinical characteristics are summarized in Table 1. Ten eyes were excluded due to recent trauma, 16 due to concomitant macular diseases, 26 due to myopia, and 31 due to incomplete follow-up. Among the 14 eyes with LMHs, seven had tractional LMHs and seven had degenerative LMHs; among the 10 eyes with VMT, four eyes had a complete outer retinal defect. Mean follow-up duration was 15.0 ± 5.7 months in the FTMH group, 13.2 ± 4.8 months in the LMH group, and 13.1 ± 6.5 months in the VMT group (*P* = 0.578).

**Table 1.** Demographic and Baseline Clinical Characteristics

Parameter	FTMH (*n* = 48)	LMH (*n* = 14)	VMT (*n* = 10)	*P*
Age (y), mean ± SD	68.4 ± 7.3	73.3 ± 7.4	73.0 ± 11.0	0.103
Sex, female (%)	50%	78.5%	80%	0.064
Pseudophakia before surgery, *n* (%)	15 (31.2)	4 (28.5)	3 (30)	0.876
BCVA pre-op (logMAR), mean ± SD	0.74 ± 0.39	0.24 ± 0.15	0.45 ± 0.14	**0.002**
BCVA post-op (logMAR), mean ± SD	0.32 ± 0.42	0.13 ± 0.11	0.26 ± 0.20	**0.004**

BCVA, best corrected visual acuity; FTMH, full thicnkness macular hole; LMH, lamellar macular hole; SD, standard deviation; VMT, vitreomacular traction.

### HF and MH Closure

Among the 48 FTMH eyes, 43 achieved successful anatomical closure, and HF displaying the characteristic vertical distribution pattern were observed in all 43 eyes at the first follow-up assessment. The five eyes with persistent MH defects were excluded from HF pattern analysis because the foveolar architecture required for vertical HF classification was absent. See a representative example in Figure 2. In contrast, HF were present in only seven of 14 LMH eyes (50%) and five of 10 VMT eyes (50%) at the first follow-up assessment. Fisher’s exact test revealed a highly significant difference in HF prevalence between the FTMH and control groups (LMH + VMT combined; *P* < 0.001). Among the five VMT eyes with HF, four had an outer retinal defect (*P* = 0.048). On the other hand, among the seven LMH eyes with HF, 28.5% were true or degenerative LMHs, and 71.4% were tractional LMHs or ERM foveoschisis (*P* = 0.141) (Fig. 2). HF were observed in 43 of 43 eyes at the first postoperative OCT (mean, 18 ± 12 days). HF preceded complete ELM restoration in nine of 43 eyes (20.9%), whereas in the remaining cases both events were detected simultaneously at the same postoperative visit. Complete ELM restoration was observed at a mean of 30.2 ± 11.2 days (range, 3–120), and EZ integrity was first documented at a mean of 6.1 ± 3.3 months (range, 1–24) postoperatively. Representative cases are shown in Figures 3^4^ to 5.

**Figure 2.**
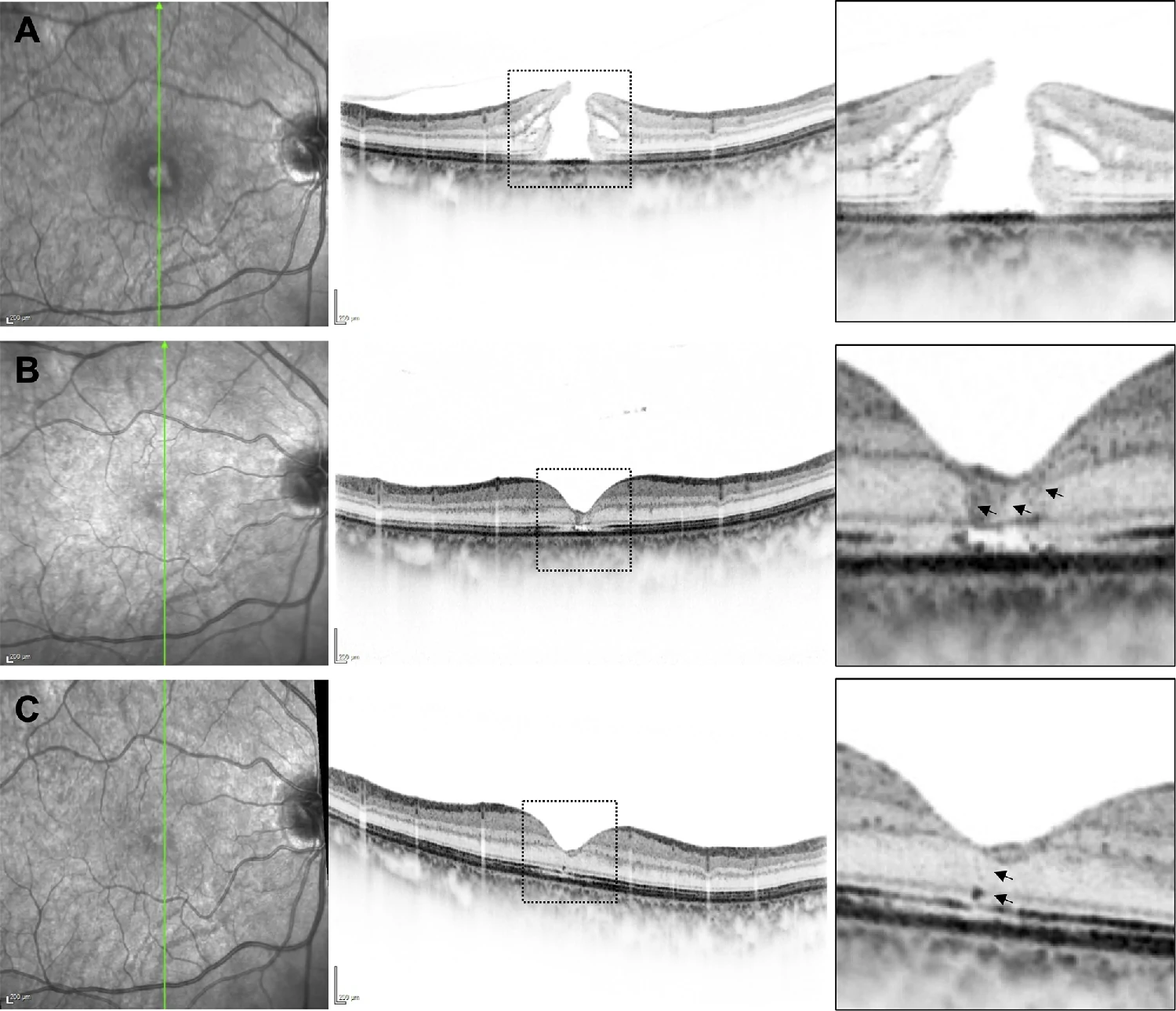
**HF with a vertical distribution pattern in the foveola after surgery for LMHs and VMT.** (**A**) Preoperative OCT B-scan of a 68-year-old male with tractional LMH (*left*), OCT B-scan at 6-month follow-up (*center*), and magnified foveolar view (*right*), demonstrating HF arranged in a vertical pattern. (**B**) Preoperative OCT B-scan of a 71-year-old female with VMT (*left*) and postoperative OCT B-scans (*center* and *right*), demonstrating an absence of HF in the foveola. (**C**) Preoperative OCT B-scan of an 81-year-old female with VMT and a complete outer retinal defect (*left*) and postoperative OCT B-scans at 4-month follow-up (*center* and *right*), demonstrating HF in a vertical pattern within the foveola (*black arrows*), with incomplete restoration of the ERM and absence of the EZ.

**Figure 3.**
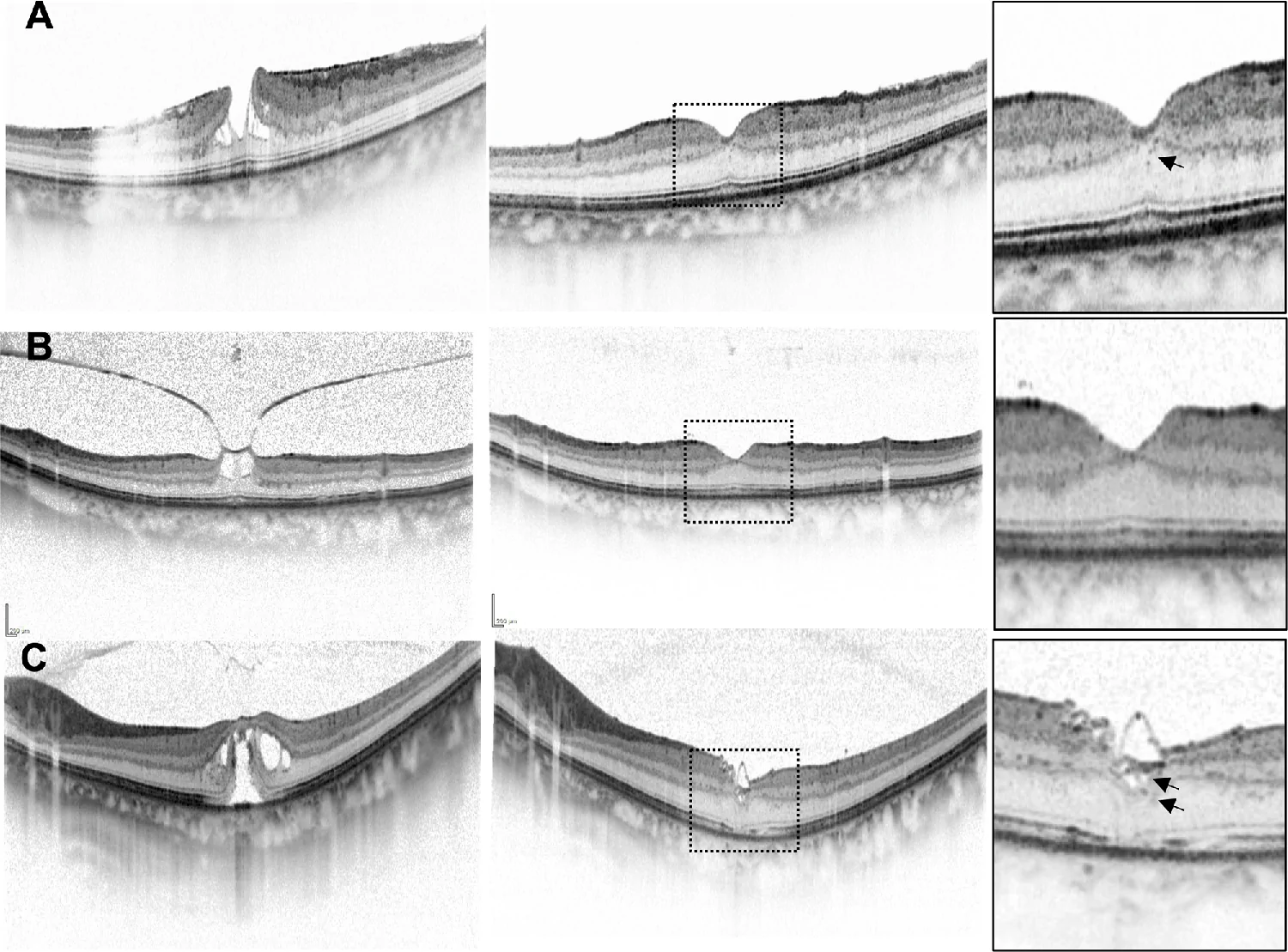
**HF with a vertical distribution pattern in the foveola and concurrent ELM restoration following surgical closure of FTMHs.** (**A**) OCT vertical B-scan in a 74-year-old female patient with a FTMH (minimum linear diameter = 321 µm). (**B**) OCT scan after 11 days from pars plana vitrectomy and ILM peeling. Note the successful closure of the MH. Magnification of the foveola is shown on the right (*white line*). Note the presence of multiple HF with a vertical distribution in the foveola. The ELM is present, but the EZ is not. (**C**) OCT scan 6 months after surgery. Note the persistence of HF in the foveola (*black arrows*), a continuous ELM, and the presence of incomplete EZ integrity.

**Figure 4.**
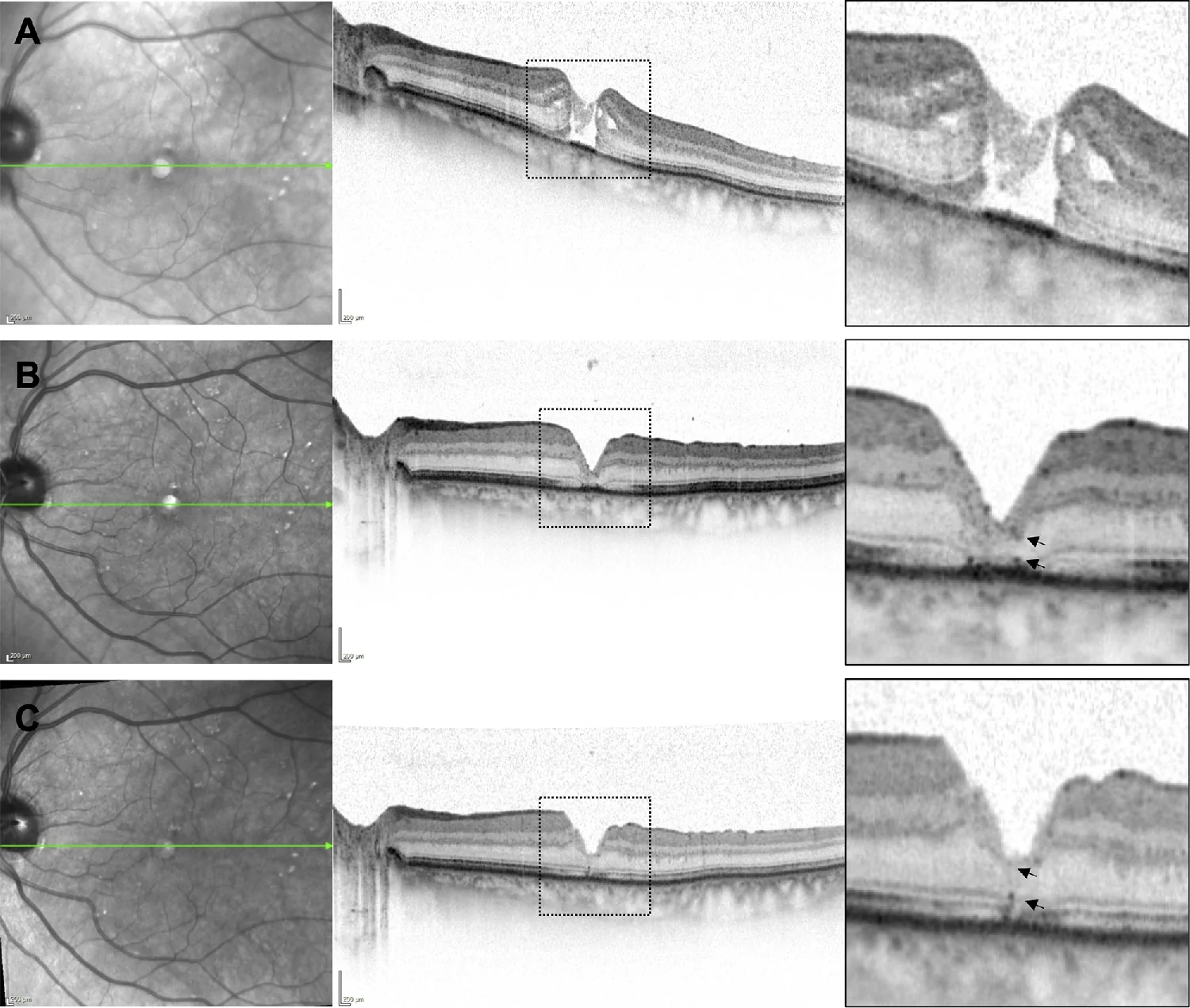
**HF with a vertical distribution pattern in the foveola preceding ELM restoration following surgical closure of FTMHs.** (**A**) OCT horizontal B-scan in a 74-year-old female patient with a FTMH (minimum linear diameter = 356 µm). (**B**) OCT scan 10 days after pars plana vitrectomy and ILM peeling. Note the successful closure of the MH. Magnification of the foveola is shown on the *right* (*white line*). Note the presence of multiple HF with a vertical distribution in the foveola. The ELM and the EZ are absent. (**C**) OCT scan 8 months after surgery. Note the persistence of HF in the foveola (*black arrows*), a continuous ELM, and the presence of incomplete EZ integrity.

**Figure 5.**
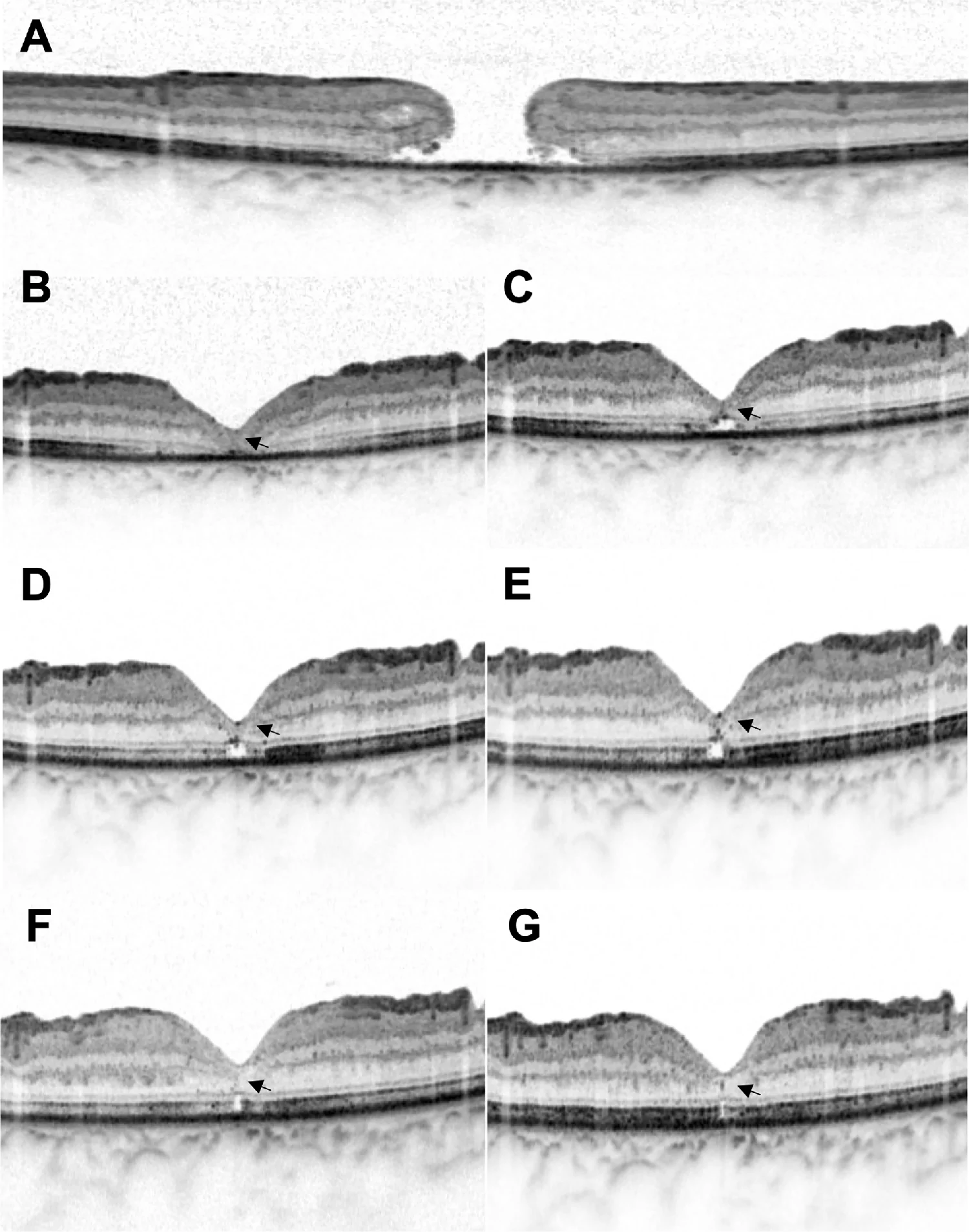
**Sequential OCT imaging illustrating the healing mechanism of a FTMH following surgery.** (**A**) Preoperative OCT vertical B-scan of a 64-year-old female with a FTMH (minimum linear diameter = 472 µm). (**B**) OCT B-scan 26 days after pars plana vitrectomy with ILM peeling, demonstrating successful hole closure, presence of HF in a vertical pattern within the foveola (*black arrows*), and absence of the ELM and EZ. (**C**) OCT B-scan at 85 days postoperatively demonstrating an increase in foveolar HF, incomplete ELM restoration, and persistent absence of the EZ. (**D**) OCT B-scan at 162 days postoperatively demonstrating persistence of foveolar HF, complete ELM restoration, and continued absence of the EZ. (**E**) OCT B-scan at 338 days postoperatively demonstrating persistence of foveolar HF, complete ELM, and early progressive formation of the EZ in the foveola. (**F**) OCT B-scan at 15 months postoperatively demonstrating a reduction in foveolar HF, complete ELM, and incomplete EZ. (**G**) OCT B-scan at 24 months postoperatively, demonstrating persistence of foveolar HF, complete ELM, and incomplete EZ integrity.

### Persistence of HF and Baseline MH Morphometry

Among all 43 successfully closed eyes, 37 eyes retained HF at final follow-up (86.0%), and six eyes showed complete resolution (14.0%). Eyes with persistent foci had significantly larger base diameter (median = 708 and IQR = 558.5–942.5 vs. median = 281 and IQR = 201.2–819.7 µm; *P* = 0.044) and lower MHI (median = 0.62 and IQR = 0.5–0.8 vs. median = 1.23 and IQR = 0.6–1.4; *P* = 0.007) compared to those with resolved foci (Table 2). Overall, both reads showed a good level of agreement in the measurements (ICC = 0.874, 95% confidence interval [CI], 0.802–0.914).

**Table 2.** Relationship Between HF Persistence and Baseline MH Morphometry

	Median (IQR)		
Parameter	Persistent HF (*n* = 37)	Resolved HF (*n* = 6)	*P*
Minimal linear diameter (µm)	286 (175.5–440.5)	172 (115.2–214)	0.426
Base diameter (µm)	708 (558.5–942.5)	281 (201.2–819.7)	**0.044**
Height (µm)	425 (370.5–524.5)	334 (286–424.7)	0.102
Index (height/base diameter)	0.62 (0.5–0.8)	1.23 (0.6–1.4)	**0.007**

BCVA, best corrected visual acuirty; EZ, ellipsoid zone; HF, hyperreflective foci.

### Final Visual Acuity, EZ Integrity, and Persistent HF

Among all 43 successfully closed eyes, 28 eyes achieved complete EZ integrity at the last follow-up (65.1%), and 15 eyes achieved incomplete EZ integrity (34.9%). On the other hand, complete ELM integrity was observed in 100% of the cases. Eyes with complete EZ integrity achieved superior final visual acuity (mean BCVA = 0.21 ± 0.16 logMAR) compared to eyes with incomplete EZ integrity (0.36 ± 0.52 logMAR; *P* = 0.038). A significant association was observed between EZ integrity and foci persistence: Among the 28 eyes with complete EZ integrity, 22 retained persistent foci (78.6%), and six eyes had resolved foci (21.4%). Conversely, among 15 eyes with incomplete EZ integrity, all 15 demonstrated persistent foci (100%), with zero eyes showing foci resolution (Fisher’s exact test, *P* = 0.047). Eyes with persistent foci showed slightly worse visual acuity (median = 0.22; IQR = 0.1–0.3 logMAR), compared to those with resolved foci (median = 0.08, IQR = 0.05–0.25 logMAR), but this difference did not achieve statistical significance (*P* = 0.234) (Table 3). Intergrader agreement for HF presence, ELM, and EZ integrity was excellent, with kappa values of κ = 0.89 (95% CI, 0.79–0.96), κ = 0.90 (95% CI, 0.80–0.96), and κ = 0.88 (95% CI, 0.76–0.93), respectively.

**Table 3.** Final Visual Acuity, EZ Integrity Status, and HF Persistence

Parameter	Persistent HF (*n* = 37)	Resolved HF (*n* = 6)	*P*
BCVA (logMAR), median (IQR)	0.22 (0.1–0.3)	0.08 (0.05–0.25)	0.234
EZ integrity (%)	22/37 (59.5)	6/6 (100)	**0.047**

## Discussion

The results of the present study suggest that intraretinal HF displaying a vertical pattern within the foveola might represent an early structural feature associated with the reparative process following successful FTMH closure, appearing during the initial phases of foveal healing and outer retinal restoration. HF were first documented at a mean of 18 ± 12 days postoperatively, at or before ELM restoration (30.2 ± 11.2 days), and EZ integrity was first recorded at a mean of 6.1 ± 3.3 months. At final follow-up, HF persisted in 86.0% of eyes and were associated with larger base diameter, lower MHI, and incomplete EZ recovery.

The interpretation of HF in the context of MH healing requires understanding the role of the Müller cell cone, a specialized structure of 25 to 35 vertical Müller cells that stabilize the central fovea by binding foveolar cone photoreceptors.^13^ Gass and colleagues^3^^,^^14^ first identified disruption of this structure as central to MH pathogenesis, and Bringmann et al.^2^ subsequently detailed its unique vertical organization and susceptibility to tractional destabilization. Scharf et al.^7^ identified a central vertical hyperreflective stress line on SD-OCT in 50% of eyes preceding FTMH formation and in 51% after surgical closure, attributing this sign to Müller cell cone reorganization. In this context, the vertical distribution pattern of HF within the foveola is compatible with an association between these OCT findings and Müller cell cone-mediated structural remodeling during MH healing. However, the cellular substrate responsible for the presence of vertical HF in the foveola cannot be determined by OCT imaging alone, as these features may arise from different components involved in tissue repair and remodeling, including cellular proliferation or migration, activated microglia, extracellular matrix changes, or alterations in the optical properties of reorganizing retinal tissue. Therefore, although Müller cell cone-mediated remodeling represents a plausible biological mechanism based on the anatomical characteristics of the foveola, future studies integrating complementary imaging modalities or histopathological analyses will be required to elucidate the biological nature of these OCT findings and their potential contribution to MH healing.

In the present study, HF appeared at or before ELM restoration in all cases, consistent with the healing hierarchy established by Bottoni et al.,^15^ who demonstrated prospectively that the ELM is the first outer retinal structure to recover after FTMH closure, becoming continuous in 53% of eyes at 1 month and 79% at 3 months, and that no eye showed EZ continuity with a disrupted ELM. The ELM represents junctional complexes between Müller cells and photoreceptors, and its early recovery depends on the integrity of the underlying glial scaffold. The observation that HF precede or coincide with ELM restoration suggests they may mark the earliest detectable phase of this process. These findings are consistent with recent findings by Francone et al.,^16^ who identified inner retinal hyperreflectivity as an early predictor of closure in FTMH eyes treated with PPV and ILM flap, using swept-source OCT under gas tamponade. However, given the retrospective design and fixed follow-up intervals of our study, the observed sequence reflects the time of first detection at scheduled visits rather than true biological onset, and further prospective studies are needed to confirm our findings.

Furthermore, the relationship between persistent HF and incomplete EZ recovery at the last follow-up further supports this sequential model. Michalewska et al.^5^ showed that photoreceptor defects decrease continuously for at least 12 months and correlate significantly with visual acuity improvement (*P* < 0.01). Bottoni et al.^15^ demonstrated that combined ELM, EZ, and outer nuclear layer recovery accounts for most visual acuity variance over 1 year. In the present series, complete EZ integrity was associated with significantly better final visual acuity (*P* = 0.038), and all eyes with incomplete EZ integrity demonstrated persistent HF (*P* = 0.047). Recently, Spaide^8^ proposed that healing of MHs after surgery involves inward migration of photoreceptors and outward migration of inner retinal layers, a pattern analogous to developmental foveation during childhood. Thus, the persistent presence of HF in eyes with incomplete photoreceptor recovery may represent an ongoing remodeling process during foveal reorganization. Furthermore, this finding may be related to the greater extent of initial foveal disruption, as persistence of HF at the last follow-up was associated with larger MH diameter and lower MHI at baseline, suggesting that a longer remodeling phase may be required in eyes with more extensive retinal injury. However, because baseline MH severity may influence both HF persistence and postoperative EZ recovery, these associations should be interpreted cautiously and considered exploratory. Further prospective studies incorporating multivariable analyses will be required to determine the independent contribution of HF persistence to postoperative structural recovery.

The association between HF persistence and hole morphometry is consistent with prior evidence linking hole size to the extent of postoperative glial activity. Oh et al.^4^ demonstrated that glial proliferation was significantly more frequent in eyes with larger basal hole diameter and lower MHI, with larger photoreceptor defects and worse visual acuity at final follow-up. Ko et al.^6^ similarly identified moderately reflective foveal lesions consistent with glial proliferation in 55% of closed FTMH eyes, with larger lesions in eyes with greater structural disruption. Our findings suggest that the extent of preoperative tissue damage may influence the magnitude and duration of the postoperative remodeling response and may partly account for the persistence of HF observed in the present study.

The inclusion of LMH and VMT eyes as comparison cohorts was aimed at evaluating whether foveolar HF were associated with the severity of baseline structural outer retinal disruption rather than representing a nonspecific postoperative OCT finding. Although LMHs and VMT have different pathophysiological mechanisms compared with FTMHs, they represent conditions characterized by variable degrees of tractional stress on the foveal architecture, with distinct involvement of the foveola and outer retinal structures. The lower HF prevalence in LMH and VMT suggests that the development of the characteristic vertical HF pattern may be related to the extent of foveolar injury, particularly in eyes showing more extensive outer retinal involvement, rather than occurring as a nonspecific consequence of surgery alone. Within the LMH group, HF were more prevalent in tractional LMHs and ERM-associated foveoschisis (71.4%) than in degenerative LMHs (28.5%), although this difference did not reach statistical significance (*P* = 0.141), likely due to the small sample size. Among VMT eyes, the association between HF and complete outer retinal defects (*P* = 0.048) further suggests that the extent of outer retinal involvement may represent an important determinant for the appearance of this OCT feature. However, given the limited sample sizes in both the LMH (*n* = 14) and VMT (*n* = 10) groups, these findings should be considered exploratory and require validation in larger prospective studies to further clarify the biological substrate of foveolar HF.

These findings might suggest a reframing of gliosis and hyperreflective lesions in retinal repair. Historically, HF and glial proliferation have been described as markers of pathological processes.^10^^,^^11^^,^^17^ However, in this retrospective series, gliosis may also represent a component of the reparative mechanism rather than solely a pathological response. The presence of HF after MH surgery may therefore represent morphological evidence of retinal repair-related remodeling. This reinterpretation aligns with contemporary understanding of wound healing, where glial and fibroblastic responses are recognized as essential for tissue repair and restoration of structural integrity.^18^ Although the retrospective design precludes establishment of causality, the present findings suggest that the presence of HF may represent an OCT feature associated with the structural reorganization occurring during MH healing.

This study has several important limitations. First, the retrospective design precludes establishment of causality, as we cannot determine whether HF actively mediate healing or merely serve as markers of the repair process. Furthermore, the small number of surgical failures (*n* = 5) limits our ability to draw firm conclusions about the relationship between absent HF and closure failure. Therefore, prospective studies with larger cohorts are needed to confirm our findings.

Second, selection bias is a significant concern. The 48.4% exclusion rate due to myopia, incomplete follow-up, concomitant macular disease, and trauma raises questions about generalizability, and eyes meeting the inclusion criteria may represent a more favorable surgical cohort than is encountered in routine practice. Furthermore, small sample sizes for the control groups (LMH, *n* = 14; VMT, *n* = 10) limit the robustness of comparative findings. Also, the exploratory nature of the statistical analyses should also be acknowledged. Given the small size of specific subgroups, the limited number of eyes available for some comparisons, and the potential impact of multiple comparisons, multivariable modeling was not feasible, and findings associated with borderline *P* values should be interpreted cautiously. Therefore, these associations should be considered hypothesis generating rather than confirmatory and require validation in larger prospective cohorts.

Third, fixed follow-up intervals mean that observed temporal relationships among HF appearance, ELM restoration, and EZ recovery reflect the time of detection at scheduled visits rather than true biological onset. Furthermore, the binary classification of ELM and EZ integrity may have limited the ability to capture the continuous and dynamic nature of outer retinal restoration after MH closure. Although this approach demonstrated excellent intergrader reproducibility, future studies incorporating quantitative OCT parameters and continuous measurements of outer retinal recovery may provide a more comprehensive assessment of the healing process. Finally, without histopathological confirmation or advanced cellular imaging, we cannot definitively establish that HF represent Müller cell cone reorganization or exclude contributions from activated microglia, infiltrating macrophages, or other glial populations.

## Conclusions

Intraretinal HF within the foveola were observed during the early postoperative phase in eyes achieving anatomical MH closure. Their detection at or before the first documented evidence of ELM restoration suggests that HF may represent an early OCT feature associated with retinal remodeling after MH closure. Persistence of HF was associated with larger baseline hole dimensions and incomplete EZ recovery, potentially reflecting a prolonged remodeling response in eyes with greater initial structural disruption. Although the retrospective design precludes causal inference, these findings support further investigation of foveolar HF as a potential imaging marker of MH healing in prospective studies. Further studies using multimodal imaging and histopathological approaches are needed to clarify the biological basis of vertically arranged foveolar HF.
